# Comorbidities and laboratory changes of sudden sensorineural hearing loss: a review

**DOI:** 10.3389/fneur.2023.1142459

**Published:** 2023-04-18

**Authors:** Wen Xie, Niki Karpeta, Busheng Tong, Yuehui Liu, Zhilin Zhang, Maoli Duan

**Affiliations:** ^1^Department of Otolaryngology, Head and Neck Surgery, The Second Affiliated Hospital of Nanchang University, Nanchang, China; ^2^Division of Ear, Nose and Throat Diseases, Department of Clinical Science, Intervention and Technology, Karolinska Institutet, Stockholm, Sweden; ^3^Department of Otolaryngology-Head and Neck Surgery, Karolinska University Hospital, Karolinska Institutet, Stockholm, Sweden; ^4^Department of Otolaryngology, Head and Neck Surgery, The First Affiliated Hospital of Anhui Medical University, Anhui, Hefei, China

**Keywords:** sudden sensorineural hearing loss, hearing loss, etiology, laboratory results, comorbidities

## Abstract

Sudden sensorineural hearing loss (SSNHL) is defined as an abrupt hearing loss of more than 30 dB in three contiguous frequencies within 72 h. It is an emergency disease requiring immediate diagnosis and treatment. The incidence of SSNHL in Western countries' population is estimated between 5 and 20 per 1,00,000 inhabitants. The etiology of SSNHL remains unknown. Due to the uncertainty of the cause of SSNHL, at present, no specific treatment targets the cause of SSNHL, resulting in poor efficacy. Previous studies have reported that some comorbidities are risk factors for SSNHL, and some laboratory results may provide some clues for the etiology of SSNHL. Atherosclerosis, microthrombosis, inflammation, and the immune system may be the main etiological factors for SSNHL. This study confirms that SSNHL is a multifactorial disease. Some comorbidities, such as virus infections, are suggested to be the causes of SSNHL. In summary, by analyzing the etiology of SSNHL, more targeting treatments should be used to achieve a better effect.

## 1. Introduction

Sudden sensorineural hearing loss (SSNHL) is defined as an abrupt hearing loss of more than 30 dB in three contiguous frequencies within 72 h (1). Associated symptoms, including tinnitus, aural fullness, sound distortion, dizziness, vertigo, and benign paroxysmal positional vertigo (BPPV), may present in some cases (2). Moreover, SSNHL patients with vertigo tend to suffer from more severe hearing loss and worse hearing recovery (3, 4) due to a higher risk of vestibular organ lesions (5).

The incidence of SSNHL in developed countries' populations is an estimated 5–20 per 1,00,000 persons per year (6). There is an overall slight male preponderance, with a male-to-female ratio of 1.07:1 (7). Regarding age distribution, Rauch demonstrated that SSNHL most frequently occurred in 43–53-year-old patients (8). On the contrary, a Japanese survey showed that SSNHL was most prevalent among patients aged 60–69 years old (9). In addition, our study showed that the peak age prevalence was in the group of patients aged 41–50 years (3).

The etiology of SSNHL remains unknown, multiple factors are suggested to be the causes of SSNHL. Some pathophysiological mechanisms, including vascular disease, viral infection, metabolic disease, autoimmunity, and combinations of multiple factors are suggested to be the causes of ISSNHL. Due to the uncertainty of the cause of SSNHL, at present, there is no specific treatment targeting the cause of SSNHL, thus resulting in poor efficacy. This brief review focuses primarily on the etiological comorbidities and laboratory changes of SSNHL. We searched the U.S. National Library of Medicine's PubMed database using the terms “sudden sensorineural hearing loss,” “sudden hearing loss,” “idiopathic sudden sensory neural hearing loss,” and “sudden deafness” as well as the keywords such as “etiology,” “risk factors,” “comorbidity,” and “laboratory results.”

## 2. Etiological comorbidities

Abundant evidence has proved that several diseases were associated with an increased risk of SSNHL; all these etiological diseases are listed in Table 1.

**Table 1:** Previous studies about the etiological comorbidities of SSNHL.

**Study design**	**Patient group (n)**	**Control group (n)**	**Etiological comorbidities [incidence (%)]^#^**	**Negative prognostic factors**	**References**
Case-control study	109	109	Hypertension (21.1)	No mention	(10)
Case-control study	141	271	Diabetes (15.6), hyperlipidemia: hypercholesterolemia (40.0),	No mention	(11)
			Hypertriglyceridemia (64.9)		
Case-control study	30	30	Diabetes (20), hyperlipidemia (20)	No mention	(12)
Case-control study	23	23	Hyperlipidemia (not described)	No mention	(13)
Case-control study	81	23	Metabolic syndrome: hypertension and hyperlipidemia (14.8)	No mention	(14)
Case-control study	181	181	Hypertension (39.2), hyperlipidemia (23.8)	Vertigo, hearing loss pattern	(15)
Case-control study	118	415	Hypertension (24), hyperlipidemia (14)	Hypertension, hyperlipidemia, diabetes, smoking	(16)
Cohort study	27,547 with depressive disorders	27,547 with anxiety disorders	Diabetes (18.45), hyperlipidemia (15.56), kidney disease (14.22)^&^	No mention	(17)
Case-control study	514	2,570	Hypertension (35.6), diabetes (19.6), hyperlipidemia (23.2)	No mention	(18)
Case-control study	3,331	13,324	Hypothyroidism (1.0): only for patients aged over 50 years	No mention	(19)
			Hyperthyroidism (2.2): only for female patients		
Retrospective cohort study	26,556	26,556	Diabetes (1.29)^&^	No mention	(20)
Prospective cohort study	73,957	73,957	Hypercholesterolemia (10.67)^&^	No mention	(21)
Retrospective cohort study	37,421 with kidney disease	37,421 without kidney disease	Diabetes (not described), kidney disease (10.24)^&^	No mention	(22)
Cohort Study	13,250 with autoimmune-disease	66,250 without autoimmune-disease	Autoimmune disease (1.09)	No mention	(23)
Cohort Study	7,619 with RA	30,476 without RA	RA (0.8)	No mention	(24)
Retrospective cohort study	464 with IDA	19,649 without IDA	IDA (1.72)	No mention	(25)
Case-control study	4,004	12,012	IDA (4.3)	No mention	(26)

RA, iron-deficient; IDA, iron deficiency anemia. ^#^For the case-control study, the incidence rate refers to the incidence rate of comorbidity in the case group; for the cohort study, the incidence rate refers to the incidence rate of SSNHL among patients with pre-existing disease. ^&^Rate: per 10,000 person-years.

### 2.1. Cardiovascular disease

#### 2.1.1. Hypertension

As shown in Table 1, hypertension is considered as one of the most common comorbidities of SSNHL. Animal experiments also showed that the blood flow in different parts of the cochlea was reduced by nearly 80% in hypertensive rats exposed to noise and 50–60% in hypertensive rats fed with an atherogenic diet (27). The cochlea is supplied by the cochlear artery, a terminal artery without any collateral vessels to compensate for any occlusion of the blood vessel. Thrombosis or vasospasm of the internal auditory artery is one of the main hypotheses to explain SSNHL. Hypertension may induce atherosclerotic changes and result in cochlear microcirculation disturbance.

#### 2.1.2. Dysrhythmia

A study elucidated that patients with dysrhythmia showed a significantly higher risk of SSNHL (28). Even after the adjustment of confounders, the incidence of SSNHL in the dysrhythmia group was higher than that in the comparison group. This finding suggests that hemodynamic instability due to dysrhythmia resulting in impaired blood perfusion to the inner ear can lead to SSNHL.

### 2.2. Metabolic disease

#### 2.2.1. Diabetes

A retrospective cohort study showed that the prevalence of SSNHL was 1.29 per 1,000 person-years among diabetic patients, which was 1.54-fold higher compared with non-diabetic subjects (20). In addition, earlier studies revealed that hearing impairment also occurred in the opposite ear, especially in high frequencies (29). Compared with diabetic patients without SSNHL, the glycated hemoglobin value was significantly higher in diabetes patients with SSNHL, and SSNHL patients with type-2 diabetes had more severe hearing loss (30). Moreover, a cohort study demonstrated that during 14 years of follow-up, a significantly lower percentage of diabetes patients with metformin use developed SSNHL compared with those without metformin intake, indicating that metformin use appeared to reduce the risk of developing SSNHL among diabetes patients (31).

Researchers found that the animal model of type-2 diabetes and obesity exhibited significantly elevated auditory brainstem response (ABR) thresholds. Regarding histological findings, outer hair cell degeneration and spiral ganglion cell loss were present in the middle and basal turns of the cochlear. This study indicates that diabetes and obesity may lead to early sensorineural hearing loss (32).

Microangiopathy may be one of the mechanisms underlying the association between diabetes and SSNHL. Other mechanisms, including upregulation of vascular endothelial growth factor, inducible nitric oxide synthase, and endothelial nitric oxide synthase, may be involved in the pathogenesis of cochlea functional loss (33).

#### 2.2.2. Hyperlipidemia

Previous studies have demonstrated that patients with SSNHL had significantly higher plasma concentrations of cholesterol, triglyceride, lipoprotein A, and low-density lipoprotein cholesterol compared with controls (34, 35).

Animal experiments revealed that after a high-fat diet for 4 months, guinea pigs' inner ears showed impaired hearing sensitivity and pathologic alterations of the cochlear, especially in the basal turn and stria vascularis (36). It has also been reported that cholesterol had different distributions among outer hair cell membranes. Furthermore, after being incubated with water-soluble cholesterol, the outer cell's lateral wall stiffness parameter increased, which impaired the activity of the outer hair cells (37). In addition, Sikora et al. found that after being fed a high-fat diet, chinchillas exposed to noise exhibited more severe hearing loss at high frequency and significantly greater hair cell loss than those in chinchillas fed with a normal diet (38).

Overall, the pathophysiological mechanism of SSNHL caused by hyperlipidemia is through the modification of the microstructure of the stria vascularis and the composition and the electromotility of the outer hair cells by elevated cholesterol, thereby increasing the cochlea's vulnerability to noise. Moreover, hyperlipidemia promotes hyperviscosity, contributes to endothelial function damage, and decreases nitric oxide release. Consequently, it promotes the formation of atheromatous plaque, which might cause occlusion of the cochlear artery, therefore resulting in SSNHL (39, 40).

### 2.3. Autoimmune diseases

According to a review written by Ralli et al. (41), sensorineural hearing loss was the most common audiovestibular symptom related to systemic autoimmune diseases. Hearing loss may be present in a sudden, slowly, rapidly progressive, or fluctuating form, and is mostly bilateral and asymmetric. SSNHL has been reported as a symptom of some systemic autoimmune diseases, such as autoimmune hepatitis, sympathetic neural hyperalgesia edema syndrome, Cogan's syndrome, systemic lupus erythematosus, multiple sclerosis, rheumatoid arthritis (RA), nodular polyarteritis, and Crohn's disease (42).

Previous studies showed that the risk of SSNHL was significantly higher in patients with antiphospholipid syndrome, multiple sclerosis, RA, and connective-tissue diseases than in patients without autoimmune diseases, and RA was in particular closely related to SSNHL (23, 24). Another retrospective study demonstrated that comorbid systemic lupus erythematosus or RA might negatively affect the prognosis of SSNHL (43). Furthermore, a systematic review reported that SSNHL could be an early manifestation of multiple sclerosis, especially in women. The pathophysiology of SSNHL caused by multiple sclerosis can be explained by the involvement of microglia attacking the central and/or peripheral auditory pathways (44).

Recently, O'Malley et al. (45) reported that the inflammatory cells are distributed in the inner ear. They found the presence of resident cochlear macrophages and the recruitment of inflammatory macrophages to the cochlea in animal models. This result indicates that the innate immune defense system of the human inner ear may involve in many otologic diseases (45).

The pathophysiology of inner ear involvement in systemic autoimmune diseases remains uncertain. The possible pathophysiology may include activated circulating antibodies against inner ear antigens, leading to antibody-dependent cell-mediated cytotoxicity; the activation of the complement system, which directly triggers cytotoxic T cells; or immune complex-mediated damage, which results in vasculitis of the inner ear and causes atrophy of the stria vascularis (46–52).

### 2.4. Hematological disorders

Hematological disorders such as aplastic anemia, sickle cell anemia, and hyperviscosity syndrome have been described as being associated with inner ear deficits. These hematological diseases may cause inner ear hemorrhage or vasculopathy (53).

#### 2.4.1. Iron deficiency anemia

A retrospective cohort study showed that children with iron deficiency anemia (IDA) demonstrated an increased likelihood of SSNHL (25). Another study also confirmed the link of SSNHL with IDA. In this study, absolute latencies for all ABR waves and interpeak latencies (except I-III interval) were significantly longer in children with IDA than in non-anemic infants (54). A population-based study also showed a significantly higher prevalence of prior IDA among participants with SSNHL compared with the controls, especially in those less than 60 years old. The researchers suggested that patients with IDA, especially those younger than 60 years, should be more aggressively surveyed to reduce hearing-related morbidities (26).

In the animal experiment, an electrophysiological study revealed that the incidence of an auditory threshold elevation of more than 15 dB was 31.85% in the iron-deficient (ID) rats, whereas it was unchanged in all the control animals. The main cochlear histopathological changes were strial atrophy and reduction of spiral ganglion cells in ID rats. So the authors concluded that the observed anomalies may be attributed solely to iron deficiency of the cochlear tissue (55).

The main cochlear pathological changes of SSNHL in ID rats were the synchronous abnormal activity of the iron-containing enzymatic, including succinic dehydrogenase and peroxidase, which in turn would disturb cell respiration and initiate peroxidative damage to the inner ear cells, resulting in a significant reduction of spiral ganglion cells and rapid damage of stereocilia of the outer and inner hair cells (56, 57).

#### 2.4.2. Leukemia

It has been reported that 16–40% of leukemia patients had otolaryngological symptoms, such as SSNHL, vertigo, tinnitus, facial paralysis, and infection (58, 59). Among hematologic malignancies, SSNHL has often been described as the initial presentation in patients with acute lymphocytic leukemia. However, recent studies have indicated that both acute and chronic leukemia were associated with SSNHL (60, 61).

Lin et al. (53) reported that during the 20 years, they had identified 14 cases of SSNHL among patients with hematological disorders, i.e., leukemia or aplastic anemia. Most of these patients presented an abnormal mean hearing level, cervical vestibular-evoked myogenic potential test, ocular vestibular-evoked myogenic potential test, and caloric test results, exhibiting a significant sequential decline in inner ear function.

Chae et al. (62) documented a case of chronic myelogenous leukemia with the first manifestation being SSNHL, and the patient's hearing was restored after leukapheresis and chemotherapy without steroids. The authors presumed that cochlear vessel occlusion as a result of elevated blood viscosity may be responsible for this patient's hearing loss.

Numerous studies have demonstrated histopathological changes in the temporal bones of patients with leukemia. These histopathological changes include leukemic infiltration, inner ear hemorrhage, infection (58, 59, 63), and hyperviscosity syndrome (64, 65).

### 2.5. Chronic kidney disease

Chronic kidney disease (CKD) can significantly increase the risk of SSNHL (17). A cohort study showed that the incidence of SSNHL was 1.57 times higher in the CKD group compared to the non-CKD group (22).

Another study reported that two patients with kidney failure suffered from profound SSNHL during the course of hemodialysis (66). Moreover, a significant decrease in cochlear microphonic and cochlear nerve action potential has been demonstrated in guinea pigs in a uremic state (67).

One possible explanation of the association between CKD and SSNHL is that the cochlea and kidney have numerous anatomic, physiological, pharmacological, and pathological similarities and have a shared antigenicity, so both are influenced by similar immunologic factors. In addition, many nephrotoxic drugs are also ototoxic. As a result, many patients with CKD may suffer from SSNHL (66, 68).

Dialysis may sometimes result in deteriorated auditory function. Rizviand and Holmes found that the endolymphatic system collapsed in patients on dialysis in a case series (69). They also found edema and atrophy in the majority of the cells of the auditory and vestibular sensory organs. A cohort study reported that hemodialysis patients with SSNHL had higher risks of hemorrhagic stroke, ischemic stroke, acute coronary syndrome, and peripheral arterial occlusive disease than hemodialysis patients without SSNHL (70).

### 2.6. Thyroid diseases

Some researchers have studied the relationship between thyroid disease and SSNHL. Nakashima et al. explored the SSNHL risk factors in a case–control study including 109 patients, reporting that patients with a history of thyroid disease had a higher odds ratio for SSNHL than those without such history (10). A case–control study with large samples showed that the correlation between hypothyroidism and increased SSNHL risk was significant only for patients aged over 50 years old and that the correlation between hyperthyroidism and SSNHL was remarkable only for female patients (19).

Thyroid autoantibodies can result in peripheral or central hearing organ dysfunction, increasing patients' susceptibility to SSNHL (71). In addition, thyroid dysfunction may lead to hypercoagulability and venous thrombosis, which may impair cochlear circulation, thus causing SSNHL (72, 73).

Overall, all these comorbidities which may affect the blood supply to the inner ear or alter the metabolism of the inner ear can cause SSNHL. Another evidence of circulatory disorder may be the main pathophysiology of SSNHL is that hyperbaric oxygen is effective for treating SSNHL. This treatment contributes to supply the oxygen needs to the peripheral neuronal structures of the inner ear (74).

## 3. Laboratory test results

In addition to hyperglycemia and hyperlipidemia, several laboratory abnormalities were reported in SSNHL patients (Table 2). The alterations of several major hematological parameters are reviewed and listed as follows.

**Table 2:** Previous studies about the laboratory findings of SSNHL.

**Study design**	**Patient group (n)**	**Control group (n)**	**Changes of laboratory outcomes in patient group**	**The meaning of the indicator**	**Negative prognostic factors**	**Reference**
Case-control study	250	250	TC, LDL, apolipoprotein B↑	Hyperlipidemia	No mention	(75)
Case-control study	30	60	TC↑, Coenzyme Q↓	Hyperlipidemia	No mention	(76)
Case-control study	29	29	TC, LDL↑, FMD↓	Hyperlipidemia and endothelial dysfunction.	No mention	(77)
Case-control study	54	55	TC, LDL↑	Hyperlipidemia	No mention	(78)
Systematic review and meta-analysis	6 articles		TC, LDL: no difference	Hyperlipidemia	No mention	(79)
Case-control study	324	972	TC, TG↑, LDL: no difference	Hyperlipidemia	No mention	(35)
Case-control study	324	972	Non-high-density lipoprotein↑	Hyperlipidemia	No mention	(80)
Case-control study	23	23	Fibrinogen, TC↑	Hypercoagulable state and hyperlipidemia	No mention	(13)
Case-control study	131	77	Blood glucose, HbA1C, lipoprotein (a), factor VIII ↑	Hyperlipidemia, diabetes and hypercoagulable state	No mention	(81)
Case-control study	118	415	Factor VIII, homocysteine↑, antithrombin, protein C↓, fibrinogen:no difference	Thrombophilia and cardiovascular risk factors	No mention	(16)
Case-control study	100	200	TC, fibrinogen, homocysteine↑, folate↓	Hyperlipidemia and cardiovascular risk factors	No mention	(82)
Case-control study	53	53	fibrinogen, erythrocyte aggregation, blood and plasma viscosity↑	Hypercoagulable state	No mention	(83)
Case-control study	142	84	Fibrinogen↑, TC, LDL, HDL: no difference	Hypercoagulable state	No mention	(84)
Case-control study	86	30	TC, TG, lipoprotein A, fibrinogen, erythrocyte aggregation, blood and plasma viscosity↑	Hyperlipidemia and hypercoagulable state	No mention	(34)
Case-control study	51	70	Blood and plasma viscosity↑	Thromboembolic factors	No mention	(85)
Case-control study	155	155	TC, homocysteine, plasminogen activator inhibitor-1, anticardiolipin↑	Cardiovascular risk factors	No mention	(86)
Case-control study	16	32	Erythrocyte filtration index↑	Microcirculation disturbance	No mention	(87)
Case-control study	30	30	FMD↓	Endothelial disfunction	No mention	(12)
Case-control study	35	35	ICAM-1, VCAM-1, E-selectin, IL-6, IL-8, and MCP-1: no different	Endothelial dysfunction	No mention	(88)
Prospective case-controlled study	37	47	VCAM-1↑	Endothelial dysfunction	No mention	(89)
Case-control study	21	21	Endothelial progenitor cells↓	Endothelial dysfunction	No mention	(90)
Case-control study	39	70	ROS, TAC, Oxidative-INDEX↑	High oxidative stress	No mention	(91)
Case-control study	43	24	Homocysteine↑, folate↓	Cardiovascular and thromboembolic risk factor	No mention	(92)
Systematic review and meta-analysis	22 articles		Folate↓	Cardiovascular and thromboembolic risk factor	No mention	(93)
Retrospective case review	203		WBC, ESR, blood glucose, HbA1C↑	Inflammation	High fibrinogen levels, WBC counts, ESR, and low FDP	(94)
Case-control study	348	537	NLR, PLR↑	Inflammation	High NLR	(95)
Case-control study	47	50	NLR, PLR, SII↑	Inflammation	High SII scores	(96)
Case-control study	60	60	NLR, PLR↑	Inflammation	High NLRs and PLRs	(97)
Case-control study	43	10	Neutrophils↑, NKCA ↓ serum levels of IL-6 ↑	Inflammation	High neutrophil counts	(98)
Prospective case-controlled study	56	56	ESR, ANA, C3, C4, and monocytes ESR↑	Immune reaction	No mention	(99)
Case-control study	64	50	HSP70, the Hsp70 bound to CIC↑	Immune reaction	No mention	(100)
Case-control study	24	24	Monocyte population, TNF-α↑	Immune reaction	No mention	(101)

TC, total cholesterol; TG, triglyceride; HDL, high density lipoprotein; LDL, low density lipoprotein; FMD, flow-mediated dilation; ICAM-1, intercellular adhesion molecule 1; VCAM-1, vascular cell adhesion molecule; WBC, white blood cell counts; HbA1C, glycated hemoglobin; ESR, erythrocyte sedimentation rate; FDP, fibrinogen degradation products; NLR, neutrophil-to-lymphocyte ratio; PLR, platelet to lymphocyte ratio; SII, systemic immune inflammation index; ANA, antinuclear antibody; Hsp70, shock protein 70; ROS, serum reactive oxygen species capacity; NKCA, natural killer cell activity; CIC; circulating immune complex; TNF-α, tumor necrosis factor-α.

### 3.1. Blood coagulation systems

Table 2 shows that laboratory abnormalities, such as hyperfibrinogenemia, antithrombin, protein C or protein S deficiency, and high factor VIII plasma levels, were associated with SSNHL. All these changes contribute to hypercoagulability and microthrombosis, which may cause cochlear ischemia and result in SSNHL.

Animal models showed increased levels of fibrinogen, accompanied by decreased cochlear blood flow as well as increased hearing thresholds. Moreover, hearing thresholds correlated negatively with cochlear blood flow (102).

Additional evidence for the role of hyperfibrinogenemia as one etiological factor of SSNHL is that acute and drastic removal of plasma fibrinogen and low-density lipoproteins can be used to effectively treat SSNHL. This treatment approach had a rapidly beneficial effect on endothelial dysfunction in SSNHL patients (103, 104).

### 3.2. Hemorheology

The changes in hemorheology observed in SSNHL patients included increased blood and plasma viscosity, erythrocyte aggregation index, and erythrocyte filtration index (Table 2). These changes can lead to impaired blood perfusion in the inner ear either by thrombosis or impaired regional blood flow.

### 3.3. Endothelial function

The biomarkers of endothelial function include flow-mediated dilation (FMD) of the brachial artery, endothelial progenitor cells (EPCs), and the expression of circulating adhesion molecules, such as soluble intercellular adhesion molecule 1 (ICAM-1) and soluble vascular cell adhesion molecule 1 (VCAM-1). Other factors, including oxidative stress, homocysteine, and folate also take part in the endothelial function.

#### 3.3.1. FMD

FMD is a simple, non-invasive, and highly repeatable method to assess endothelial function. The mechanism of FMD is that after compression of the brachial artery for some minutes, the increased blood flow can induce shear stress, which can activate the endothelium to release nitric oxide with the consequence of vasodilation. This phenomenon can be monitored by ultrasonography. Diminished FMD is an early sign of subclinical atherosclerosis and is associated with coronary atherosclerosis (105–107). Recently, researchers also found reduced FMD among SSNHL patients (12, 77).

#### 3.3.2. EPCs

EPCs are circulating cells, and their properties are similar to embryonal angioblasts. They can differentiate into mature endothelial cells. Increased EPCs have been found in case of acute vascular damage such as limb ischemia, acute myocardial infarction, or vascular trauma. By contrast, decreased EPCs were linked to a higher incidence of cardiovascular events (108, 109).

By analyzing peripheral blood CD34^+^KDR^+^CD133^+^ cells, researchers found that the circulating levels of EPCs were much lower in SSNHL patients compared with controls (90). The results of this study confirm the existence of endothelial dysfunction in SSNHL patients.

#### 3.3.3. Circulating adhesion molecules

Increased expression of some molecules is the early evidence of endothelial dysfunction. The activated endothelial cells can increase the expression of soluble ICAM-1 and soluble VCAM-1, and these molecules can mediate leukocyte adhesion to the endothelium and activate atherosclerosis formation (89, 110).

One prospective case–control study showed higher ICAM-1 and VCAM-1 in SSNHL patients (89). However, inconsistent results have been documented by another study, indicating that there was no difference between ICAM-1 and VCAM-1 between SSNHL patients and the controls. The authors considered that the role of soluble adhesion molecules in the pathogenesis of SSNHL remained unclear and needed further investigation (88).

#### 3.3.4. Oxidative stress

The balanced reactive oxygen species (ROS) and antioxidant system can maintain the normal physiological oxidative status in living organisms. On the contrary, the imbalance between ROS and total antioxidant capacity is thought to be a potential pathogenetic mechanism leading to endothelial dysfunction. If excessive ROS are not buffered by the cellular antioxidants, they can react with cellular macromolecules and promote lipid peroxidation, which may cause DNA damage and induce protein and nucleic acid modifications (111).

Recent studies have reported a significantly higher ROS in SSNHL patients, as well as oxidative stress status, supporting the vascular impairment involvement in ISSNHL etiopathogenesis (91, 112). The microcirculation disturbance due to an ischemic event may relate to increased oxidative stress, which may synergistically account for endothelial damage, especially in terminal microvascular systems (91, 113).

Other findings also reflect the involvement of oxidative stress in SSNHL. In a successive pioneering study, Cadoni et al. described an association between SSNHL and low serum levels of the antioxidant Co-enzyme Q (CoQ) (76).

#### 3.3.5. Homocysteine and folate

Hyperhomocysteinemia is considered to be a cardiovascular and thromboembolic risk factor for atheromatous and vascular events (114). Homocysteine can promote platelet aggregation, hypercoagulability, oxidative stress response, endothelial impairment, and smooth muscle cell proliferation (115).

As an important regulator of homocysteine, folate is a coenzyme necessary for one-carbon metabolism. Low levels of folate may contribute to increased plasma levels of homocysteine (92). Lower serum folate and higher homocysteine levels have been found among SSNHL patients than among controls (92).

In general, it is known that endothelial dysfunction has a primary role in regulating vascular tone by modifying lipoproteins, thrombogenesis, and transformation of circulating monocytes into foam cells (82). Moreover, it can counterbalance pro-aggregation and anti-aggregation properties or even regulate coagulation conditions by mediums such as heparin. If endothelial dysfunction exists, the blood supply to the inner ear will be disturbed because of the sudden and transient thrombotic event, which could explain the nature of SSNHL (77, 92).

## 4. Inflammation

Chronic inflammation may lead to microvascular damage and atherogenesis, which increases ischemic risk in a direct way (116). Several studies revealed that some biomarkers of inflammation, including white blood cell (WBC) counts, neutrophil count, neutrophil-to-lymphocyte ratio (NLR), platelet-to-lymphocyte ratio (PLR), systemic immune inflammation index (SII) values, tumor necrosis factor-α level, and monocyte population were higher in SSNHL patients compared to the control groups. By contrast, lymphocyte count was significantly higher in the control group (Table 2). The lower NLR level might be taken into account as a novel potential marker to predict a better prognosis. A meta-analysis including 12 retrospective cohort studies also confirmed that NLR might be a useful biomarker to determine the onset and prognosis of SSNHL (95).

The high WBC counts among SSNHL may reflect an immune response to inner ear damage induced by ischemic changes or infections (94). In addition, the interrelation between neutrophils and endothelium may contribute to increased damage to the endothelium and was reported to explain platelet adhesion in patients with unstable angina (117). An elevated platelet count leading to an increased PLR might therefore lead to an increase in vascular endpoints. The SII, which is defined as platelets × neutrophils/lymphocytes, can serve as a prognostic marker for malignancies and inflammatory conditions. According to Ulu et al., as a novel index, the SII can be an indicator of SSNHL and it can predict the prognosis of SSNHL (96).

Masuda et al. (98) recruited 43 patients with SSNHL and found that, in SSNHL patients, neutrophils were above the reference range, natural killer cell activity (NKCA) was low and serum levels of interleukin-6 (IL-6) were higher compared to controls. Moreover, neutrophil count level was correlated with more severe hearing loss and a worse prognosis. The authors hypothesized that high neutrophils together with low NKCA and high IL-6 may activate nuclear factor-κB in the cochlea and lead to SSNHL (98).

## 5. Immune system

As shown previously, immune factors are involved in the onset of SSNHL. Studies have found elevated levels of Circulating Immune Complexes and Heat Shock Proteins 70 in SSNHL patients, as well as IgG antibodies against the inner ear-specific proteins cochlin and β-tectorin (100, 118). These findings have provided compelling evidence that antibody-mediated tissue damage and Type III immunocomplex-mediated immune reaction in the inner ear are the pathogenetic mechanisms of the development of SSNHL. In addition, Baradaranfar M. reported that mean erythrocyte sedimentation rate, antinuclear antibody, C3, C4, and monocytes were higher in the case group (99).

In addition, no matter what kind of administration method, the use of steroids greatly improved the recovery of hearing in patients with SSNHL (119). The beneficial effect of corticosteroids in SSNHL could be due to an immunosuppressive and anti-inflammatory effect.

## 6. Conclusion

SSNHL is a multifactorial disease and its underlying mechanism remains uncertain. Some etiological comorbidities involving multiple systems may play a role in its pathogenesis. Atherosclerosis, microthrombosis, inflammation, and immune system may be the main etiological factors of SSNHL. In summary, by analyzing the etiology of SSNHL, more targeting treatments should be directed at the underlying cause to achieve a better effect.

## Author contributions

Based on discussions with all authors, WX drafted the manuscript, which all authors revised. All authors contributed to the study design. All authors approved the final version submitted for publication.
